# In Silico Screening and In Vivo Evaluation of Potential CACNA2D1 Antagonists as Intraocular Pressure-Reducing Agents in Glaucoma Therapy

**DOI:** 10.3390/ph14090887

**Published:** 2021-08-31

**Authors:** Hanxuan Li, Mohamed Moustafa Ibrahim, Hao Chen, Wei Li, Monica M. Jablonski

**Affiliations:** 1 Department of Pharmaceutical Sciences, University of Tennessee Health Science Center, Memphis, TN 38163, USA; hli97@uthsc.edu (H.L.); hchen55@uthsc.edu (H.C.); 2 Department of Ophthalmology, Hamilton Eye Institute, University of Tennessee Health Science Center, Memphis, TN 38163, USA; mibrahi2@uthsc.edu; 3 Department of Pharmaceutics, Faculty of Pharmacy, Mansoura University, Mansoura 35516, Egypt

**Keywords:** glaucoma, intraocular pressure, CACNA2D1 antagonists, in silico screening, homology model, molecule docking

## Abstract

Glaucoma is a leading cause of permanent vision loss and current drugs do not halt disease progression. Thus, new therapies targeting different drug targets with novel mechanisms of action are urgently needed. Previously, we identified CACNA2D1 as a novel modulator of intraocular pressure (IOP) and demonstrated that a topically applied CACNA2D1 antagonist—pregabalin (PRG)—lowered IOP in a dose-dependent manner. To further validate this novel IOP modulator as a drug target for IOP-lowering pharmaceutics, a homology model of CACNA2D1 was built and docked against the NCI library, which is one of the world’s largest and most diverse compound libraries of natural products. Acivicin and zoledronic acid were identified using this method and together with PRG were tested for their plausible IOP-lowering effect on Dutch belted rabbits. Although they have inferior potency to PRG, both of the other compounds lower IOP, which in turn validates CACNA2D1 as a valuable drug target in treating glaucoma.

## 1. Introduction

Glaucoma is a group of eye diseases that can slowly and asymptomatically steal human sight. Most glaucomatous patients do not recognize that they may have glaucoma until they suffer significant vision loss. Vision field loss associated with glaucoma occurs due to optic nerve damage that results mainly from the persistent pressure exerted by elevated IOP, especially in case of primary open angle glaucoma (POAG). POAG accounts for more than 90% of glaucoma cases all over the world and is considered to be one of the leading causes of irreversible blindness, especially in elderly people [1]. Because of this, decreasing IOP is considered as the first-line therapeutic solution in the management of glaucoma. Several commercial IOP-lowering products are available in the drug market. Unfortunately, none of these therapies cure POAG and most of them are associated with local and systemic side effects [2,3,4]. Furthermore, some of them suffer from diminishment of the pharmacological effect upon repeated applications (i.e., tachyphylaxis), which is considered as a fatal defect, especially in case of medication used in treatment of chronic disease such as POAG [5]. In addition, most of these IOP-lowering medications have a short duration of action that require several daily doses, which could exaggerate the severity of their side effects. Even the newly FDA-approved medications that are intended to be used once daily are associated with severe local and systemic side effects that result in poor patient compliance and acceptance. Examples of the side effects of recently approved products are Vyzulta (latanoprostene bunod, 0.024%), which is associated with a permanent pigmentation of the eyelids, lashes and iris [6]; and Rhopressa (netasudil, 0.02%), which causes conjunctival hyperemia and hemorrhage, eye pain upon instillation and cornea verticillate [7]. In addition to the previously mentioned side effects, the chronic use of topically applied anti-glaucoma medication may result in a disturbance of the tear film that impacts the health of the patient’s ocular surface and results in a glaucoma-related ocular surface disease and dry eye condition [8]. Therefore, identification of safe, effective and long-acting drug molecules that can selectively interact with a specific target site inside the eye and have the ability to control the IOP is still an urgent medical need. In our recent publications, we demonstrated the localization of a subunit of the L-type calcium channel—CACNA2D1—in the ciliary body and trabecular meshwork, which are the tissues responsible for production and drainage of the aqueous humor, respectively, of human, rabbit and mouse eyes. We reported that pregabalin (PRG) can target this protein and reduce IOP by decreasing the production and/or increasing the drainage of aqueous humor [9,10].

In drug discovery, molecular docking is a commonly used method to predict the binding mode and affinity of a ligand to a protein with a known structure. Typically, the ligand will be tried to dock against a specific region of the protein. The best binding pose will then be determined, and a docking score will be calculated typically based on the energy reduction of the two entities before and after the docking. In this situation, a lower docking score indicates a higher energy reduction and thus a tighter binding between the ligand and the protein [11]. When the structure of the protein is unknown, which is the case in our study, to perform molecular docking, the structure of the protein will need to first be predicted. Homology modeling is considered the most accurate among the computational structure prediction methods for this purpose [12]. In this method, a template of that protein, which is the sequence of another protein with a known structure, must first be identified and selected. The higher identity between the sequence of those two proteins usually means that a more reliable homology model will be constructed. The two sequences will then be aligned and corrected, and a homology model will then be built based on the structure of the template protein and experimental data. Post-modification of the model is sometimes needed for better results.

In the current study, to further validate our target protein—CACNA2D1—as a potential target for the treatment of glaucoma, we used the docking method to identify several compounds that are structurally similar to PRG with a good affinity to the target protein. These compounds were also tested in vivo to validate their therapeutic effect.

## 2. Results and Discussions

### 2.1. Homology Model of CACNA2D1 and In Silico Screening

**Figure 1:**
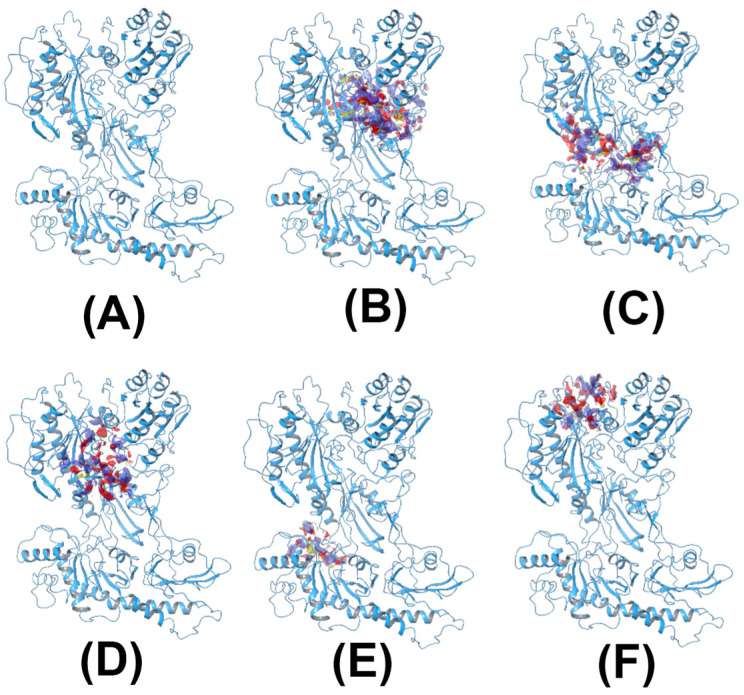
The homology model of CACNA2D1 (**A**) and the corresponding binding sites identified by SiteMap ((**B**–**F**) Sites 1–5, respectively). The yellow mesh in the binding sites indicates the hydrophobic map, while the blue and red colors indicate the hydrogen-bond donor and hydrogen-bond receptor maps, respectively.

**Figure 2:**
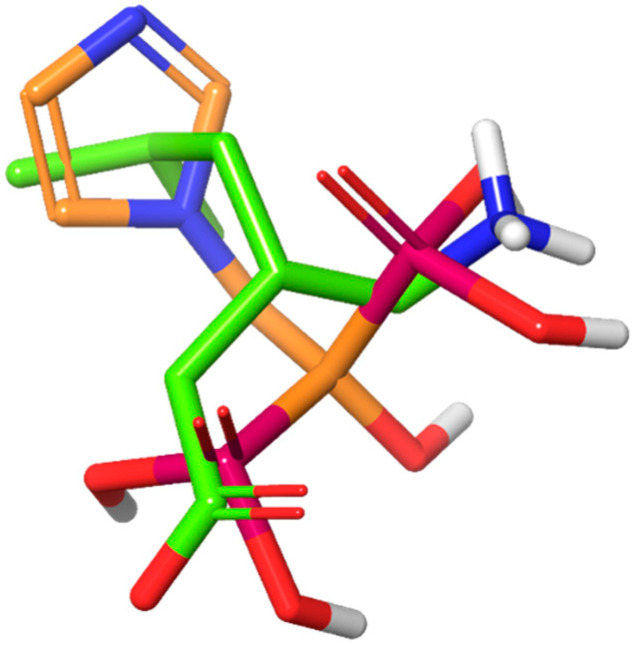
The overlay of the binding pose of PRG (green) and zoledronic acid (gold).

**Figure 3:**
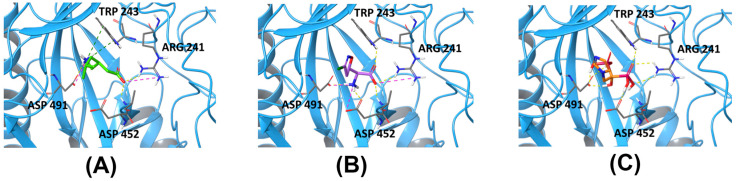
The binding poses of PRG (**A**), acivicin (**B**) and zoledronic acid (**C**) at Site 3 of CACNA2D1. Yellow dash lines indicate hydrogen bonding, pink dash lines indicate salt bridges and the green ones indicate a pi–cation interaction.

To screen for the hits of CACNA2D1, a homology model was built and five possible binding sites were identified (Figure 1). PRG was then used to dock against the five sites to understand its binding mode. The binding affinities of PRG differs dramatically among the five binding sites. The docking scores were −3.585 kcal/mol, −2.909 kcal/mol, −10.016 kcal/mol, −5.616 kcal/mol and −4.245 kcal/mol for Sites 1 to 5, respectively. Although compounds from the NCI library were docked against all five sites, because the docking score of PRG to Site 3 is superior to other sites, the binding affinity to Site 3 became our primary consideration. Acivicin, identified from the compound library, had a docking score of −12.222 kcal/mol, which was the highest of the compounds we evaluated. Zoledronic acid, although it had a much lower docking score of −6.867 kcal/mol, was considered as another hit, because its binding pose aligns well with that of PRG (Figure 2). The binding poses of all the above-mentioned compounds are illustrated in Figure 3.

As previously reported, PRG is a promising IOP-lowering medication that targets CACNA2D1 [9,10]. We predicted that acivicin and zoledronic acid, both of which have hypothetically similar binding modes as PRG, would possess a similar IOP-lowering activity. Thus, to validate our hypothesis and the evaluate our model, acivicin and zoledronic acid were both tested in vivo regarding their efficacy and safety compared to PRG. Acivicin is a glutamine analogue antibiotic produced as a fermentation product of *Streptomyces sviceus*, which has an antitumor activity [13]. Zoledronic acid is an FDA-approved medication for treatment of osteoporosis or as adjunct medication in cancer chemotherapy as an intravenous infusion (Reclast^®^, Aclasta^®^ and Zometa^®^) [14].

### 2.2. Preparation of Viscous Eye Drops Containing 0.6% w/v of Different Drug Molecules

Because these drug molecules that we identified are soluble in an aqueous vehicle, they can rapidly drain from the eye surface upon topical application into the eye. For this reason, 0.2% *w*/*v* Carbopol 981 viscous eye drops were selected as the vehicle for all the tested molecules, including PRG. Carbopol 981 is characterized by its reasonable viscosity, biocompatibility and bioadhesiveness [15,16]. Carbopol 981 is considered one of the most safe crosslinked polyacrylic acid derivatives due to the absence of benzene solvent residues [17]. Bioadhesion is a very important parameter for a topically applied ophthalmic formulation, allowing it to adhere to the eye surface (cornea and conjunctiva) and prevents its rapid drainage, either outside the eye or through the nasolacrimal duct. Moreover, the viscosity of Carbopol 981 prevents the immediate washout of the formulation from the eye surface, thus allowing enough time for the bioadhesion reaction to occur [10,18].

### 2.3. pH Measurement of Different Eye Drops

The measured pH of the tested viscous eye drops (Table 1) ranged between 4.9 and 5.5, which are in the pH range that could be easily tolerated by the natural eye buffering system without causing any discomfort [19].

### 2.4. In Vivo IOP-Lowering Efficacy Evaluation of Different Eye Drops after a Single Dose Application

**Figure 4:**
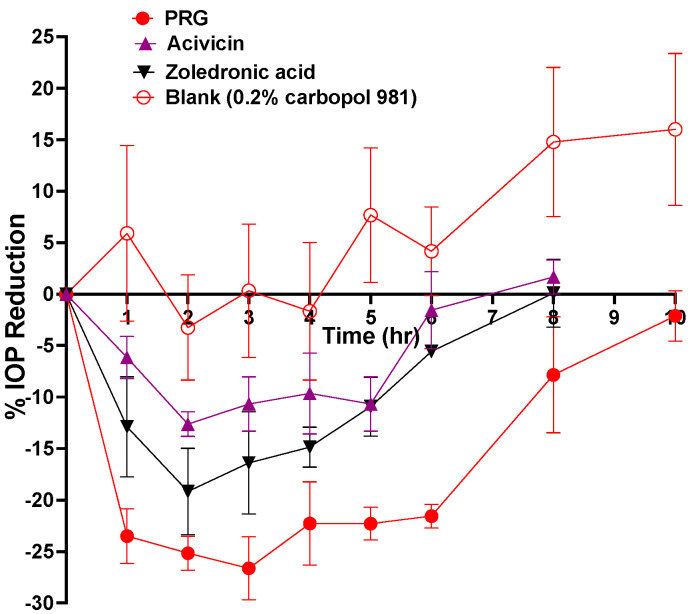
% IOP reduction of DB rabbits (*n* = 3) after topical application of 100 μL of viscous eye drops containing 0.6% *w/v* of each compound in 0.2% *w/v* Carbopol 981. The PRG and blank data presented with permission from ACS Nano [10].

The abilities of these molecules to lower the IOP were tested on Dutch belted rabbits and compared to the IOP-lowering effect of PRG in the same animal model. The IOP-lowering results of all the tested molecules are plotted in Figure 4 and the pharmacodynamic parameters are listed in Table 1. The IOP-lowering efficacy of the different molecules can be arranged as follow: PRG > zoledronic acid > acivicin (Table 1 and Figure 4). Although acivicin and zoledronic acid demonstrate inferior potency compared to PRG, their similar IOP-lowering activity indicates a shared mode of action with PRG (Figure 4).

### 2.5. In Vivo Safety Evaluation of Different Eye Drops after a Single Dose Application

**Figure 5:**
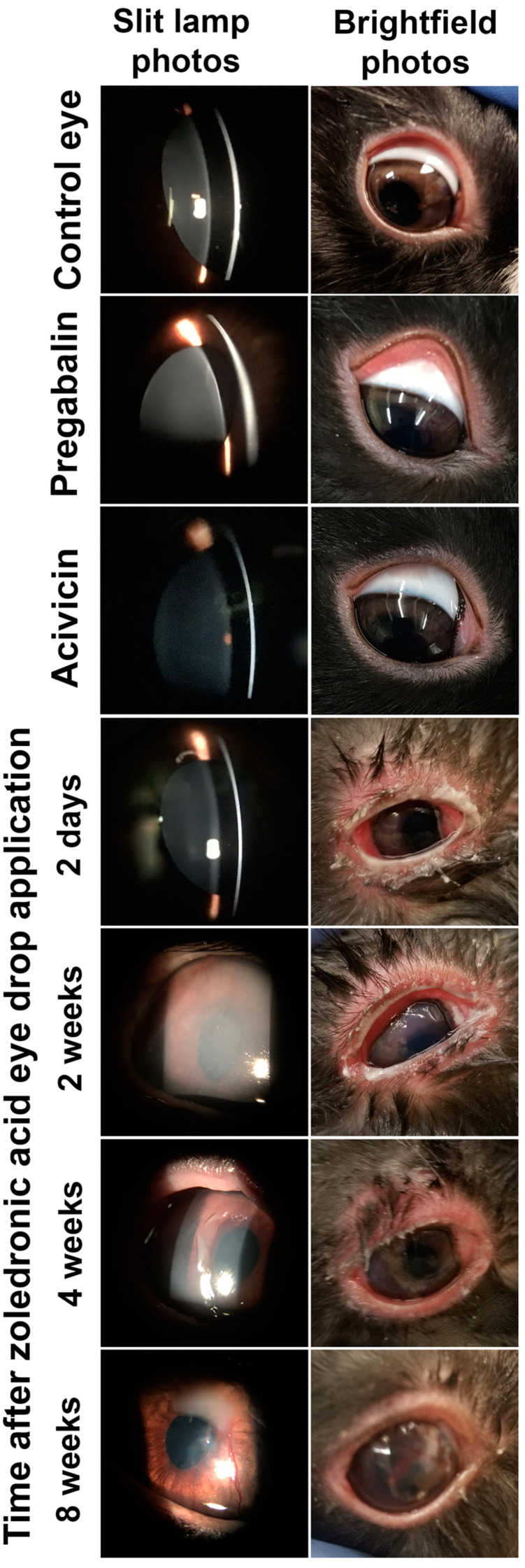
In vivo safety evaluation of eye drops containing different molecules after a single topical application. Zoledronic acid eye drop caused eye reactions up to eight weeks after application. Control eyes refer to the contralateral eye of rabbits that received zoledronic acid eye drops. Photos of the control eyes and those dosed with pregabalin and acivicin were taken 2 days after topical application of a single eye drop of the corresponding formulation.

Regarding the safety of these new molecules as potential IOP-lowering therapies, neither PRG nor acivicin show any signs of irritation, toxicity or allergic reactions (Figure 5). In contrast, zoledronic acid showed severe eye toxicity reactions that have been started 2 days after the topical application of zoledronic acid eye drops. All rabbits receiving zoledronic acid eye drops did not show any problem during the first 24 h after the application. During the second day after application, all rabbits started to show toxic side effects in the treated eyes, such as redness, tearing and thick ocular discharge. As suggested by the veterinary doctor at our university, after the toxicity sign started to appear, treatment was started with isotonic boric acid eyewash and erythromycin antibiotic eye ointment twice a day. With time, the severity of the side effects were exaggerated and new toxicity signs started to appear. The peak of the side effects occurred two weeks after the application, with all rabbits’ eyes developing corneal swelling and the appearance of a white membrane that covered all the eye surface (cornea and conjunctiva). Subsequently, the inflammation began to decrease. Unfortunately, a new permanent inflammatory condition (corneal vascularization) appeared in all eyes dosed with zoledronic acid. After two months of treatment with isotonic boric acid eyewash and erythromycin eye ointment all toxicity signs disappeared except for corneal vascularization (Figure 5). Although Nourinia et al. have reported the safety of zoledronic acid after intravitreal injection into the eyes of pigmented rats [20], our in vivo safety study confirmed the ocular toxicity of the zoledronic acid molecules. There are two case reports that are in agreement with our finding, which demonstrated the occurrence of severe eye inflammation following intravenous infusion of a zoledronic acid solution [21,22].

## 3. Materials and Methods

### 3.1. Materials

PRG (≥97% purity) and triethanolamine were purchased from Sigma Aldrich (St. Louis, MO, USA). Carbopol 981 was obtained as a gift sample from Lubrizol Advanced Materials, Inc. (Cleveland, OH, USA). Acivicin (NSC163501) was purchased from Cayman Chemical (Ann Arbor, MI, USA). Zoledronic acid (NSC721517) was purchased from AK Scientific Inc. (Union City, CA, USA).

### 3.2. Animals

Dutch belted rabbits, mixed males and females, aged 5–7 months, weighing 1.5−2.5 kg, purchased from Covance Inc. (Princeton, NJ, USA), were used to test the IOP-lowering effects of the tested molecules. All animals were examined before the study and appeared free of any clinically observable abnormalities. All rabbit eyes were healthy with no injury or history of injury. The IOP difference between the two eyes of the same rabbit did not exceed 1 mmHg. Through the whole study, rabbits had free and continuous access to food and water. All procedures including rabbits were previously approved by the Animal Care and Use review board of the University of Tennessee Health Science Center (UTHSC), Memphis, TN. The protocol also followed the Association of Research in Vision and Ophthalmology (ARVO) Statement for the Use of Animals in Ophthalmic and Vision Research and the guidelines for laboratory animal experiments (Institute of Laboratory Animal Resources, Public Health Service Policy on Humane Care and Use of Laboratory Animals).

### 3.3. Methods

#### 3.3.1. Homology Model of CACNA2D1

Because no CACNA2D1 crystal structure has been published to date, we built a homology model and used it for the docking studies based on the procedure described as follows: The protein sequence of CACNA2D1 was obtained from the UniProt database and was subsequently used for the online BLAST search. The voltage-gated calcium channel Ca(v)1.1 (PDB: 5GJV [23]) with 91% sequence identity was identified and used as the template to build the homology model using Prime. The non-template loops with less than 7 residues were subsequently refined using the VSGB solvation model and OPLS3 force field. The model was further prepared by using the protein preparation wizard in Maestro during which the ionization state at pH 7.0 ± 2.0 was generated using the Epik module, which was then used without further modification.

#### 3.3.2. Virtual Screening

The binding sites of the abovementioned homology model of CACNA2D1 were identified using SiteMap and the top five sites were used for docking studies. The structure of PRG was first prepared by Ligprep with the OPLS3e force field and was subsequently used to dock against the five sites. Similarly, ligands from National Cancer Institute (NCI) database were also prepared and docked against the same 5 sites.

#### 3.3.3. Preparation of Viscous Eye Drops Containing 0.6% *w*/*v* of Different Drug Molecules

Because all the used drug molecules are soluble in aqueous media, they could be easily prepared in the form of aqueous solutions. Unfortunately, these aqueous solutions could be rapidly drained from the eye surface before exerting any pharmacological response. For this reason, we incorporated these molecules in bioadhesive viscous Carbopol 981 eye drops. Carbopol 981 in a concentration of 0.4% *w*/*v* was soaked in Milli-Q water overnight until full swelling. PRG and acivicin were dissolved separately in Milli-Q water, while zoledronic acid was dissolved in 1% *w*/*v* triethanolamine solution, at a concentration of 1.2% *w*/*v*. Equal volumes of 0.4% *w*/*v* Carbopol 981 gel and drug aqueous solutions were mixed together using a vortex mixer to produce the final bioadhesive viscous eye drops, which were kept protected from light in closed air-tight containers at 5 °C until the time of use. After mixing, the final products were composed of 0.6% *w*/*v* of each drug in 0.2% *w*/*v* Carbopol 981 gel. To ensure sterility of the final products, all the used tools and water were sterile and all procedures were performed under aseptic conditions.

#### 3.3.4. pH Measurement of Different Eye Drops

The pH of the prepared eye drops was measured according to our previously published protocol [10]. A gram of each formulation was diluted in 20 mL Milli-Q water and mixed well using a magnetic stirrer and subjected to pH measurement by a pH meter (Corning pH meter 440; Corning Inc., Corning, NY, USA). The measurement was done in triplicate and the results presented as the mean ± SEM.

#### 3.3.5. In Vivo Evaluation of Different Eye Drops after a Single-Dose Application

1. Efficacy evaluation

The IOP-lowering effect of the different eye drops containing different drug molecules was evaluated using Dutch belted rabbits (*n* = 3) according to our previously published protocols [10]. Each rabbit was given 100 μL of each eye drops into the inferior conjunctival sac of one eye, while the other eye was given 100 μL of the blank formulation (i.e., 0.2% *w*/*v* Carbopol gel vehicle) and used as a control. A Tono-pen AVIA (Reichert Technologies, Depew, NY, USA) was used to measure the IOP. The baseline IOP was measured immediately before application of the eye drops and then was taken hourly until it returned back to its baseline value. At each time point, three readings were taken and averaged for each eye. The IOP measurement was repeated until we measured a fixed value at each reading. The relative efficacy of each drug molecule was evaluated by comparing the calculated pharmacodynamic parameters of the three eye drops containing the different drug molecule. The calculated pharmacodynamic parameters include maximum percent IOP reduction; the time required to reach the maximum decrease in percent IOP (T_max_); the time required for IOP to return back to its baseline (i.e., end of drug effect; T_end_); and the total area under the percent IOP reduction-versus-time curve (AUC). All pharmacodynamic calculations were carried out using GraphPad Prism-9 software (GraphPad Software Inc., San Diego, CA, USA). All results were expressed as the mean ± SEM.

2. Safety evaluation

The biosafety of the tested drug molecules after acute ocular exposure was tested on the eyes of Dutch belted rabbits (*n* = 3) according to our previously published protocols [18]. Rabbit eyes were visually examined for any problems or abnormalities before the application of the eye drops. Each rabbit was placed in rabbit restrainer (Plas Labs Inc., Lansing, MI, USA) to prevent them from touching their eyes at the beginning of the study (for 4 h). Each rabbit received 100 μL of each eye drops into the inferior conjunctival sac of one eye, while the other eye received 100 μL of the blank formulation (i.e., 0.2% *w*/*v* Carbopol gel vehicle) and used as a control. All eyes were visually evaluated for the appearance of irritation, toxicity or allergic reactions, such as tearing, inflammation, corneal swelling, conjunctival redness, hyperemia or swelling, etc., after 1, 2, 3, 4, 6, 8, 24, 48 and 72 h after the application. In addition to the visual evaluation, slit-lamp examinations were performed after 1, 2, 3 and 7 days. More examinations were performed to evaluate any irritation, toxicity or allergic reactions.

## 4. Conclusions

Glaucoma is one of the leading causes of irreversible blindness. Current drugs suffer from diminishment of activity and/or severe side effects upon prolonged usage. To address these problems, a new target, CACNA2D1, was identified, and PRG, which targets the protein, was found and showed a promising IOP-lowering effect in our previous studies. To further evaluate CACNA2D1 as a potential drug target, several other molecules binding to this protein were identified and tested for their in vivo activity.

In our current study, to identify additional compounds that bind to CACNA2D1, a homology model was built and used to dock against the NCI library to find possible hits. Two compounds, namely, acivicin and zoledronic acid, stood out among the others and became the best candidates. This model was validated after testing these compounds in vivo for their IOP-lowering effect. Our results demonstrate that though they have lesser potency, these compounds share the same target protein and binding sites as PRG. Together, these data suggest CACNA2D1 is a validated drug target in treating glaucoma and worthy of further investigation for more potent compounds.

## Figures and Tables

**Table 1:** pH of the viscous eye drops containing different molecules and the pharmacodynamic parameters after topical application of a single dose of different viscous eye drops to Dutch belted rabbits.

Parameters	PRG	Acivicin	Zoledronic Acid
pH	5.3 ± 0.11	4.9 ± 0.15	5.5 ± 0.21
T_max_ (h)	2.33 ± 0.67	2.67 ± 0.67	2.33 ± 0.88
T_end_ (h)	9.33 ± 0.67	7.33 ± 0.67	8.0 ± 0.00
%IOP reduction at T_max_	28.98 ± 1.8	13.46 ± 2.03	22.06 ± 2.5
AUC %.h	170 ± 16.4	52.16 ± 8.7	82.47 ± 10.8

## Data Availability

Not applicable.
